# A Cross-Sectional Study on the Characteristics of Physical Activity in Pre-Frail Older Adults

**DOI:** 10.3390/ijerph182312328

**Published:** 2021-11-24

**Authors:** Motoaki Takamura, Toshimasa Sone, Takayuki Kawamura, Reiko Suzuki, Nobuaki Moriyama, Seiji Yasumura

**Affiliations:** 1 Faculty of Health Sciences, Tohoku Fukushi University, Sendai 981-8522, Japan; k-taka@tfu-mail.tfu.ac.jp; 2 Department of Public Health, School of Medicine, Fukushima Medical University, Fukushima 960-1247, Japan; moriyama@fmu.ac.jp (N.M.); yasumura@fmu.ac.jp (S.Y.); 3 Department of Occupational Therapy, School of Health Sciences, Fukushima Medical University, Fukushima 960-1247, Japan; sone-t@umin.ac.jp; 4 YOBOU-FUKUSHI Health Promotion Center, Tohoku Fukushi University, Sendai 981-8522, Japan; s-reiko@tfu-mail.tfu.ac.jp

**Keywords:** pre-frailty, physical activity, moderate-to-vigorous physical activity, self-initiated citizen group exercise activities

## Abstract

This cross-sectional study aimed to characterize the physical activity (PA) of older adults with pre-frail status by examining sedentary behavior (SB) and PA using triaxial accelerometer data, with non-frail older adults as the control group. In this study, we divided the study participants into older adults who regularly participated in self-initiated citizen group exercise activities and those who did not. Data were collected between September and December 2017. We analyzed data from 256 older adults (women: 86.3%) aged ≥65 years. The interaction effect of participation status (participation and non-participation group) and frailty status (pre-frail and non-frail group) for moderate-to-vigorous PA (F = 9.178, *p* = 0.003) and daily mean number of steps (F = 9.351, *p* = 0.002) was significant. For the participation group, there was no difference between pre-frail older adults and non-frail older adults regarding length of SB and PA time, indicating that PA level was maintained in the participating pre-frail older adults. In contrast, moderate-to-vigorous PA and daily mean number of steps were low in pre-frail older adults who did not participate in the activities. The opportunity to participate in self-initiated group exercise activities and other PAs in the community may help pre-frail older adults maintain their PA.

## 1. Introduction

Recent studies on frailty in older adults have been conducted in a wide range of fields and have examined the physical problems associated with frailty, such as sarcopenia [1,2] and falls [3]. These studies have found that physical frailty in particular is a condition resulting in lifestyle dysfunction caused by a decline in motor function due to aging, and is related to subsequent prognosis, with a high proportion of older adults transitioning to needing support and nursing care [4,5,6] and having increased mortality risk [7,8].

Frailty has been described as “a state of reduced ability to recover from stress resulting from an age-related decline in reserves” [9]. The most widely used definition of frailty is the frailty phenotype proposed by Fried et al. [10], using a set of five physical phenotypic components: unintentional weight loss, exhaustion, weakness, slow walking speed, and low physical activity with a biological basis. Among these criteria, an individual is classified as non-frail when none of the components are applicable to them, pre-frail when one to two items are applicable, and frail when three or more items are applicable. Although only a few reports show the findings for older adults in pre-frail status separately from their physical frailty status, Makizako et al. [6] report that physical frailty, even when pre-frail, significantly impacts the risk of future disability.

Various risk factors have been associated with older adults’ progression to frailty, such as prolonged sedentary lifestyle and decline in the amount of physical activity (PA) [11,12]. Frailty is considered reversible and a state in which functioning can be maintained or improved with appropriate intervention and support [13,14,15]; studies have shown the beneficial effects of various intervention programs. Recent evidence on sedentary behavior (SB) and PA is divided regarding approaches for reducing frailty risk, with some studies considering reducing sedentary time and replacing it with low-intensity physical activity (LPA) as the best approach [16,17] and some, others advocate moderate-to-vigorous physical activity (MVPA) [18,19]. However, intervention has a limited effect in older adults with severe frailty [20,21]. Many previous studies have compared non-frail or pre-frail older adults and frail older adults and shown reduced PA in frail older adults [22,23], with a clear focus on frailty status, but very few studies have reported detailed characteristics of older adults with pre-frail status [3,24].

Furthermore, the practice and effects of various intervention programs have been reported as measures to prevent frailty. A systematic review by Apostolo et al. [25] reported that group physical exercise programs improved frailty, but the evidence to make the same claim for individual exercise was insufficient. In Japan, which is transitioning into a super-aged society, proactive efforts are underway to prevent the need for long-term care, including measures against frailty [6,26]. Among these types of intervention programs, providing a place for exercise and interaction through self-initiated citizen group exercise activities, conducted mainly by local residents, has been effective in preventing frailty [27]. However, previous studies have not clarified the differences in PA between pre-frail and non-frail older adults based on whether they participate in self-initiated senior citizen group exercise activities. Therefore, this study aimed to elucidate the PA of older adults with pre-frail status by examining SB and PA, with non-frail older adults as the control group and to examine the characteristics of PA in older adults showing varying early frailty statuses (non-frail vs. pre-frail older adults) and to analyze the potential impact of participation in self-initiated citizen group exercise activities.

## 2. Materials and Methods

### 2.1. Study Design and Participants

This study used a cross-sectional design. The target area of this study was the town of Yamamoto, in Miyagi Prefecture, Japan. As of 2017, the total population of Yamamoto was 12,469, and there were 4718 individuals aged ≥65 years (aging rate, 37.8%). The study participants were older adults aged ≥65 years who also participated in self-initiated citizen group exercise activities, run in 19 locations in the town, by invitation from the Yamamoto Community General Support Center and in senior club networking events held in three districts of the town.

In this study, the participants were divided into two groups: those participating in the self-initiated group exercise activities formed the participation group, and those who gathered at the senior club networking events formed the non-participation group. Older adults who participated in both activities were included in the participation group. Because the subjects who participated in both activities had more opportunities to exercise than the subjects who participated in the senior club networking events, they were assigned to the self-initiated group exercise participation group. The inclusion criterion was: ability to independently walk and perform basic activities of daily living, while the exclusion criteria were: participants certified as disabled (Long-term care level 1–5) by the Long-Term Care Insurance [28], presence of severe underlying illnesses, and difficulty in communicating. The inclusion and exclusion criteria were assessed through a questionnaire from which the participants were asked questions, while ensuring participant safety, and compliance with ethical standards.

The research objectives, body measurements, questionnaire survey, and accelerometer requirement were verbally explained to all participants, and a written informed consent was obtained from each. The survey was approved by the ethical review committee of Tohoku Fukushi University on 27 July 2017 (No. RS170708) and Fukushima Medical University on 12 September 2017 (No. 29174).

### 2.2. Physical Frailty Criteria

This study used the Japanese version of the Cardiovascular Health Study (J-CHS) [6,29,30,31], an improved version of the frailty phenotype (CHS criteria) proposed by Fried et al. [10] as an indicator of frailty, modified to better suit the Japanese population. Frailty was determined based on the following five items: (1) shrinking (unintentional weight loss of up to 3 kg in the past six months); (2) weakness (hand grip strength: male <26 kg, female <18 kg); (3) exhaustion (affirmative answer to the question “Did you feel exhausted without any reason in the last two weeks?”); (4) slowness (walking speed <1.0 m/s); and (5) low PA (answer “No” to both questions, “How many days per week do you engage in light exercise or calisthenics?” and “How many days per week do you engage in regular exercise or sport?”). The participants were classified as non-frail when none of the items were applicable to them, pre-frail when one to two items were applicable, and frail when three or more items were applicable [10,29]. Participants who were determined to be frail were excluded from the analysis.

### 2.3. Questionnaire Survey

All surveys were conducted between September and December 2017. Information on the participants’ basic characteristics, including age, sex, educational history, whether they lived alone, current alcohol consumption, and smoking, was collected using a self-administered questionnaire. Information on past and present history of lower back pain, knee pain, stroke, hypertension, heart disease, diabetes mellitus, osteoporosis, and arthropathy was also collected in the questionnaire (Supplementary File).

### 2.4. Physical Function and Performance

All surveys were held at open venues, such as meeting rooms and public halls, in each district in the town, and the measurement sessions were performed by well-trained staff who assisted participants in filling out the questionnaires, measured physical fitness, and explained the accelerometer survey. The participants’ height and weight were measured, and their body mass index was calculated. Walking speed was measured twice by recording the participants’ normal walking speed over a distance of 5 m [32,33,34], and the walking speed (m/s) was the mean of the two measurements. Grip strength was measured using a digital grip strength dynamometer (TKK 5401 Grip-D; Takei Scientific Instruments, Tokyo, Japan) [30,35]. The measurement was performed in standing position, and the mean was calculated based on two measurements. Standing on one leg with eyes open and arms on the hips was performed twice using the leg on which the participants found it easiest to stand, and the maximum time of the two measurements was used. The maximum time limit was 60 s, and if the subject reached 60 s in the first measurement, the second measurement was not obtained [36,37].

### 2.5. SB and PA Assessment

SB and PA times were measured using a triaxial accelerometer (Active style Pro HJA-750C; Omron Healthcare, Kyoto, Japan). The validity of the Active style Pro in estimating activity intensity has been confirmed [38,39]. The participants were requested to wear the accelerometer on their waist for seven consecutive days from when they woke up in the morning until they went to bed at night and could remove it only when bathing or swimming. During the survey, to minimize the possibility of PA being promoted by wearing the accelerometer, it was programmed such that only the clock was displayed on the device [40]. The accelerometer epoch length was set to 1 min [41]. The accelerometer data usage criterion was defined as non-wearing time when the accelerometer had no signal (estimated intensity of ≤0.9 metabolic equivalent units (METs)) for ≥60 min (with allowance for up to 2 min of some limited movement (≤1.0 METs)) [17,41,42]. The participants who wore the device for ≥10 h a day (600 min) and recorded valid data for ≥4 days (including one day off) in seven consecutive days were included in the analysis [43,44]. Individual PA was based on the activity intensity evaluated from the data recorded on the accelerometer, with activity of ≤1.5 METs defined as SB, 1.6–2.9 METs as light-intensity PA (LPA), and ≥3.0 METs as moderate-to-vigorous PA (MVPA) [45].

### 2.6. Statistical Analyses

The mean value and standard deviation were calculated for accelerometer wearing time, SB time, LPA time, and MVPA time. The ratio (%) of SB and PA time to wearing time was also calculated.

The *t*-test and Mann–Whitney U-test for continuous scale were used to compare the basic attributes of pre-frail and non-frail older adults, and the χ^2^ test or Fisher’s exact test for nominal scale was used for intergroup comparison. To examine the interaction effect of frailty status (pre-frail and non-frail) and participation status (participating and non-participating groups), a two-way analysis of variance (ANOVA) was performed by setting frailty status and participation as independent variables, and SB, LPA, MVPA, and the number of steps as dependent variables. When an interaction was observed, a simple main effect test was performed by the Bonferroni method. In addition, we performed analysis of covariance (ANCOVA) adjusted for age, sex, accelerometer wearing time, alcohol consumption, lower back pain, knee pain, and cardiovascular disease as covariates. The distribution of time spent in MVPA and the number of steps per day were positively skewed; therefore, we used a logarithmic transformation before the analysis. The descriptive tables display non-transformed data for easy interpretation.

Furthermore, considering the possibility of “low physical activity” items among the frailty criteria affecting the results, we confirmed this action using sensitivity analysis. First, we excluded the “low physical activity” item from the five items and reclassified those who did not correspond to any of the four items as non-frail, and those who corresponded to one or two items as pre-frail. Then, we performed a two-way ANOVA and ANCOVA, as mentioned earlier, to confirm the effect of this item on the outcome. In addition, due to differences in the number of men and women in the pre-frail and non-frail groups, trends were confirmed using a sensitivity analysis targeting women. All statistical analyses were performed using SPSS Statistics for Windows (version 23.0; IBM Corp., Tokyo, Japan). Statistical significance was set at *p* < 0.05.

## 3. Results

**Figure 1:**
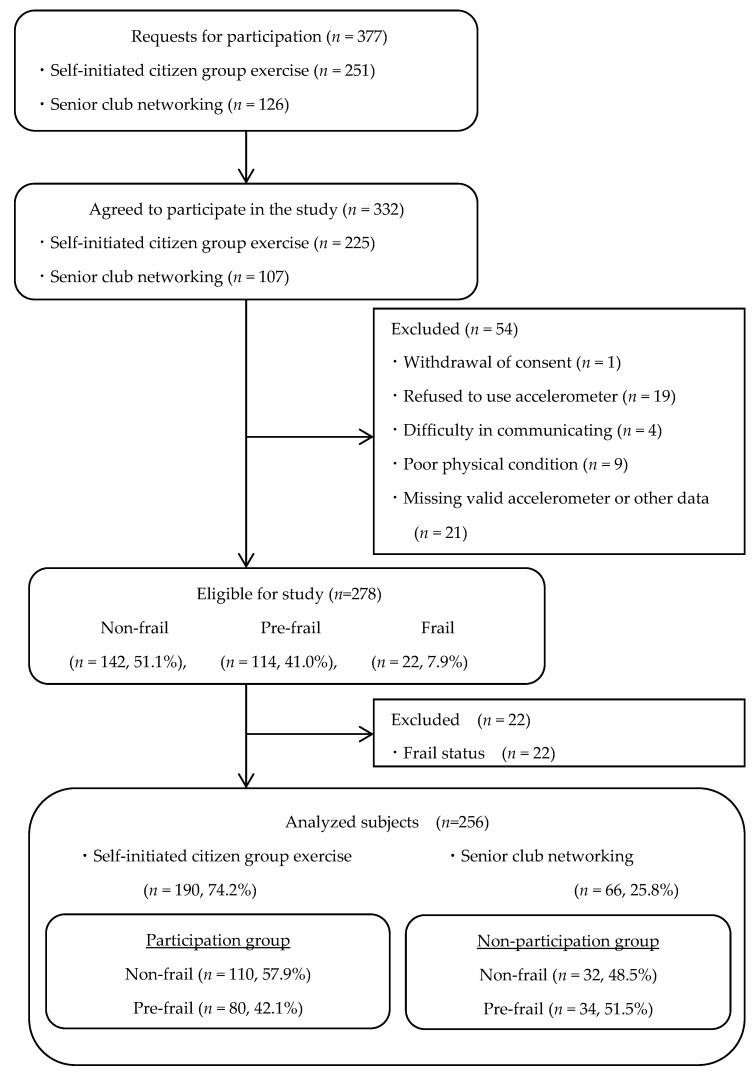
Participant selection flow-chart.

Consent was obtained from 332 individuals against requests sent to 377 individuals for cooperation with survey. A total of 54 individuals were excluded 19 persons refused to use accelerometer, one withdrew consent, 21 had missing valid accelerometer or other data, four had difficulty communicating, and nine were in poor physical condition. In addition, the nine persons who were in poor physical condition were those who reported experiencing difficulty in wearing the accelerometer due to symptoms such as cold, lower back pain, and so forth during the 7-day accelerometer-wearing period. The prevalence of pre-frailty among the remaining 278 participants according to the J-CHS criteria was 41.0% (*n* = 114), that of non-frailty was 51.1% (*n* = 142), and that of frailty was 7.9% (*n* = 22). Thus, an additional 22 individuals with frailty were excluded, and the final number of participants in the analysis set was 256 (non-frail, *n* = 142 (55.5%); pre-frail, *n* = 114 (44.5%)). Of them, 190 (74.2%) participated in self-initiated citizen group exercise activities, and 66 (25.8%) did not (Figure 1).

In the participation group, 80 were pre-frail (42.1%) and 110 non-frail (57.9%). The percentage of women was 91.3% in the pre-frail group and 90.9% in the non-frail group, and the mean ages were 74.1 ± 5.7 years and 73.2 ± 4.7 years, respectively, with no significant difference. In the non-participation group, 34 were pre-frail (51.5%) and 32 non-frail (48.5%).The percentage of women was 79.4% in the pre-frail group and 65.6% in the non-frail group, and the mean ages were 80.1 ± 7.1 years and 77.3 ± 5.7 years, respectively, with no significant difference. The percentage of participants with heart disease (26.5%) was significantly higher in the pre-frail group (*p* = 0.028). Table 1 presents the characteristics of the participants in the analysis.

Table 2 shows the results of the two-way ANOVA and ANCOVA. The interaction effect of frailty status and participation status for both MVPA and number of steps (MVPA, F = 12.760, *p* < 0.001; steps, F = 11.954, *p* < 0.001) was significant. Further, the main effect of participation status (participation vs. non-participation) was significant (MVPA, F = 10.854, *p* = 0.001; steps, F = 8.626, *p* = 0.004) and the main effect of frailty status (non-frail vs. pre-frail older adults) was significant (MVPA, F = 12.323, *p* < 0.001; steps, F = 15.232, *p* < 0.001).

The results of the simple main effect test showed significantly lower MVPA and step scores in the pre-frail older adults from the non-participation group than in the non-frail older adults (MVPA, F = 17.004, *p* < 0.001; steps, F = 18.364, *p* < 0.001). No significant difference was observed in pre-frail older adults from the participation group compared to the non-frail older adults (MVPA, F = 0.004, *p* < 0.952; steps, F = 0.189, *p* < 0.664). Further, comparing the pre-frail older adults in the participation group with the non-participation group, significant decrease in MVPA and step scores was found in the pre-frail older adults in the non-participation group (MVPA, F = 23.134, *p* < 0.001; steps, F = 20.061, *p* < 0.001).

Even after adjusting for covariates by ANCOVA, the interaction effect of frailty status and participation status for both MVPA and number of steps (MVPA, F = 9.178, *p* = 0.003; steps, F = 9.351, *p* = 0.002) was significant.

Furthermore, considering the possibility of low PA items among the frailty criteria and the difference in the number of men and women in the pre-frail and non-frail groups affecting the results, trends were confirmed using a sensitivity analysis (data not included here).

## 4. Discussion

This study aimed to characterize the PA of older adults with pre-frail status by examining SB with non-frail older adults as the control group, and to analyze the potential impact of participation in self-initiated citizen group exercise activities.

The results showed that in the non-participation group, the pre-frail older adults were a little older than the non-frail older adults, and a larger proportion had physical ailments, such as heart disease and knee pain. The presence of such related ailments has been found to raise the risk of future onset of frailty [6,7,30,46,47,48,49,50]. In addition, the results concerning PA in pre-frail older adults in this study revealed that MVPA and steps were still significantly lower in this group than in non-frail older adults, even after adjusting for these physical ailments. This is a very important finding, in that it indicates the minor changes in PA occurring between non-frail and pre-frail statuses; there has been almost no such discussion on this point in previous studies. Nagai et al. [19] also reported that, compared to the non-frail and pre-frail groups, SB time was significantly higher and LPA time and MVPA time significantly lower in the frail group. Based on these results, it is assumed that not only will MVPA and steps decline further for pre-frail older adults in the non-participation group, but that LPA time will decrease, and SB time will increase, leading to frail status. Many studies [11,12] have examined PA decrease with SB increase and occurrence of frailty; thus, it is important to detect changes in MVPA and steps in the pre-frail status at an early stage.

In contrast, in the participation group, there was no significant difference in SB or PA between the pre-frail and non-frail groups, and there were no characteristic differences in MVPA or steps as was observed in pre-frail older adults in the non-participation group. Previous studies have shown that PA in older adults with pre-frail status is almost the same as that in non-frail older adults [19,51]. Although the results of the current study are consistent with these findings, our results indicate that PA in pre-frail older adults is maintained at about the same level as PA in non-frail older adults, specifically in the participation group. Based on data regarding SB and PA in non-frail older adults from previous studies, SB is estimated at 48–56%, LPA at 41–47%, and MVPA at 3–5% of daily awake time [19,44,51]. In the current study, pre-frail older adults had low SB (48.9%), high LPA (44.7%), and an MVPA of 6.4%; therefore, we considered them active. The self-initiated group exercise was held once a week, with the residents gathering for a 90 min multicomponent exercise program incorporating balance, step, and stretching exercises and various games. Participants usually perform moderate-intensity dumbbell exercises using a light dumbbell load of 300–700 g. For people with pre-frail status in this study, participating in regular self-initiated citizen group exercise activities may have led to opportunities to maintain PA levels.

There are various reasons that may contribute to pre-frail older adults maintaining their PA in the self-initiated exercise group. The first is related to activity intensity. It is not practical for frail older adults to take on more than a moderate-to-vigorous intensity load, and opinions are divided on recommending exercise at the LPA level [16,17] versus the MVPA level [52]. Yamada et al. [27] suggested that the usefulness of participation in community-based self-management groups requires appropriate selection of exercise load intensity depending on frailty status. Further, studies have reported that the length of time spent on activities at, or above, moderate intensity increases as a quadratic function of the number of steps, indicating a strong relationship [13,14]. Considering these previous findings, it is important for pre-frail older adults to set PA at or above moderate intensity and to have the opportunity to walk and engage in other activities. Although it is possible that pre-frail older adults have secured sufficient activity intensity through self-initiated exercise group activities, further research is needed to clarify the detailed PA needs of pre-frail older adults.

The second reason for PA remaining steady is that, participating in such organized activities may be leading to opportunities for the participants to go out regularly and establish a connection with society through interaction with group members. Fujita et al. [53] showed that leaving the house less than once a week increased the risk of developing a gait disorder at an odds ratio of 4.02 in a two-year follow-up study, compared to leaving the house one or more times a day. Nemoto et al. [54] reported that social group engagement affects total PA, moderate-intensity PA, and brisk walking in older adults. These studies support the idea that in addition to maintaining and improving physical function through exercise, the group exercise activities helped participants in the current study connect with the community by providing them the chance to go out once a week and by facilitating social interaction [26], which may also have been useful in maintaining PA. Therefore, programs such as the self-initiated citizen group exercise activities and other community PAs may be useful initiatives for maintaining and preventing the decline of PA in pre-frail older adults.

The present study has several limitations. First, most of the participants were women, and the participation group was larger than the non-participation group. In addition, we assumed that those in the participation group were highly motivated and had high physical functioning from the start. Therefore, although bias adjustment and stratified analysis were performed, the results’ representativeness and generalizability to community-dwelling older adults as a whole is extremely limited. Second, because this was a cross-sectional observational study, it is not possible to determine the causal relationship between participation in self-initiated group exercise activities and maintenance of PA or between pre-frailty status and reduced PA. Third, this study focused on understanding the characteristics of physical frailty and PA. For this reason, consideration of the evaluation of social, mental, and psychological factors, which may have affected frailty and PA, was insufficient. In future, a wide range of perspectives should be incorporated to understand and interpret the contributing factors in detail. Fourth, although the accelerometer can objectively evaluate activities, it does not clearly distinguish between sitting and standing postures, nor does it sufficiently reflect underwater activities, such as bathing and swimming, or activities involving stepping up and down, such as ascending or descending stairs. Therefore, it cannot be ruled out that the overall SB and PA levels may have been underestimated.

Despite these limitations, this study has several strengths. We compared the PA of pre-frail and non-frail older adults by stratifying them into older adults who participate in self-initiated citizen group exercise activities and those who do not. By doing so, we were able to clarify the PA characteristics of pre-frail older adults. Understanding these characteristics of pre-frailty PA, detecting minor changes related to pre-frailty, and linking these observations to action would be beneficial for frailty prevention.

## 5. Conclusions

The MVPA and number of steps were significantly lower in pre-frail older adults who did not participate in group exercise activities than in pre-frail older adults who participated in these activities, and there was no difference in length of SB or PA time between participating pre-frail older adults and non-frail older adults, indicating maintenance of PA level. Therefore, participating in self-initiated citizen group exercise activities and other PAs in the community may help maintain pre-frail older adults’ PA levels. Further studies are needed to reveal various PA characteristics among pre-frail older adults to facilitate the development of preventive countermeasures at a community level.

## Figures and Tables

**Table 1:** Participant characteristics.

	Participation Group			Non-Participation Group		
	Non-Frail	Pre-Frail	*p*-Value	Non-Frail	Pre-Frail	*p*-Value
	*n* = 110	*n* = 80		*n* = 32	*n* = 34	
Age (years)	73.2 ± 4.7	74.1 ± 5.7	0.302	77.3 ± 5.7	80.1 ± 7.1	0.092
Sex, women	100 (90.9)	73 (91.3)	0.935	21 (65.6)	27 (79.4)	0.209
BMI (kg/m^2^)	23.6 ± 3.2	23.6 ± 3.2	0.871	24.0 ± 3.5	24.5 ± 3.2	0.558
Education (junior or senior high school/vocational college, university)	20 (18.2)	13 (16.3)	0.442	4 (12.5)	4 (11.8)	1.000
Living alone (yes)	13 (11.8)	16 (20.0)	0.729	2 (6.3)	8 (23.5)	0.084
Alcohol consumption (yes)	38 (34.5)	20 (25.0)	0.158	11 (34.4)	2 (5.9)	0.004
Smoking (yes)	2 (1.8)	4 (5.0)	0.241	1 (3.1)	0 (0.0)	0.485
Physical function						
Hand grip strength (kg)	24.3 ± 4.8	22.5 ± 6.7	0.006	26.1 ± 5.7	22.1 ± 6.1	0.002
Usual walking speed (m/s)	1.34 ± 0.17	1.28 ± 0.20	0.019	1.27 ± 0.19	1.12 ± 0.21	0.003
One leg standing (s)	42.5 ± 20.2	38.7 ± 21.9	0.137	36.3 ± 21.6	22.6 ± 21.3	0.011
Pain and primary disease						
Low back pain (yes)	40 (36.4)	39 (48.8)	0.087	16 (50.0)	18 (52.9)	0.811
Knee pain (yes)	30 (27.3)	29 (36.3)	0.187	9 (28.1)	17 (50.0)	0.069
Stroke (yes)	0 (0.0)	1 (1.3)	0.421	0 (0.0)	3 (8.8)	0.239
Hypertension (yes)	56 (50.9)	36 (45.0)	0.421	19 (59.4)	26 (76.5)	0.136
Heart disease (yes)	10 (9.1)	4 (5.0)	0.287	2 (6.3)	9 (26.5)	0.028
Diabetes mellitus (yes)	10 (9.1)	11 (13.8)	0.312	3 (9.4)	5 (14.7)	0.710
Osteoporosis (yes)	18 (16.4)	17 (21.3)	0.391	6 (18.8)	6 (17.6)	0.908
Arthropathy (yes)	27 (24.5)	23 (28.7)	0.516	5 (15.6)	11 (32.4)	0.113
Components of physical frailty criteria						
Exhaustion, *n* (%)	-	36 (45.0)		-	15 (44.1)	
Weight loss, *n* (%)	-	29 (36.3)		-	8 (23.5)	
Weakness, *n* (%)	-	21 (26.3)		-	10 (29.4)	
Slowness, *n* (%)	-	6 (7.5)		-	10 (29.4)	
Low physical activity, *n* (%)	-	4 (5.0)		-	8 (23.5)	
Acc wearing time (min/day)	904.5 ± 89.6	870.2 ± 83.9	0.008	875.4 ± 95.2	835.3 ± 84.4	0.075

Note: Data are shown as mean ± standard deviation or % (*n*). Student’s *t*-test and Mann–Whitney U-test were used for continuous scale, and χ^2^ test or Fisher’s exact test for nominal scale. BMI: body mass index. Acc: accelerometer.

**Table 2:** A comparison of the times spent in sedentary behavior (SB) and physical activity (PA) between factors.

				2-Way ANOVA		ANCOVA
			Main Effect	Main Effect	Interaction	Interaction
	Participation Group	Non-participation Group	Participation/ Non-Participation	Non-Frail/ Pre-Frail		
	Mean ± SD	Mean ± SD	F (*p*-Value)	F (*p*-Value)	F (*p*-Value)	F (*p*-Value)
SB (min/day)						
Non-frail	460.1 ± 103.8	443.4 ± 89.6	0.365 (0.546)	0.506 (0.477)	3.166 (0.076)	2.539 (0.112)
Pre-frail	424.7 ± 97.8	458.6 ± 94.5				
LPA (min/day)						
Non-frail	389.9 ± 90.4	375.6 ± 98.7	4.407 (0.037)	1.017 (0.314)	1.011 (0.316)	0.656 (0.419)
Pre-frail	389.8 ± 88.6	349.3 ± 91.3				
MVPA (min/day)						
Non-frail	54.5 ± 36.2	56.4 ± 37.5	10.854 (0.001)	12.323 (<0.001)	12.760 (<0.001)	9.178 (0.003)
Pre-frail	55.7 ± 36.9	27.4 ± 21.3				
Steps (steps/day)						
Non-frail	5552.5 ± 3325.8	5625.4 ± 3063.1	8.626 (0.004)	15.232 (<0.001)	11.954 (<0.001)	9.351 (0.002)
Pre-frail	5090.0 ± 2266.9	3202.8 ± 1842.7				

Note: Analyses were performed using 2-way ANOVA and ANCOVA adjusted for age, sex, accelerometer wearing time, alcohol consumption, lower back pain, knee pain and cardiovascular disease. SB: sedentary behavior; LPA: light-intensity physical activity; MVPA: moderate-to-vigorous physical activity; Steps: daily mean number of steps. 2-way ANOVA: 2-way analysis of variance; ANCOVA: analysis of covariance.

## Data Availability

The data presented in this study are available on request from the corresponding author. The data are not publicly available due to privacy or ethical restrictions.

## Supplementary Materials

The following are available online at https://www.mdpi.com/article/10.3390/ijerph182312328/s1, Supplementary file: Physical Activity Survey.
