# Supplementary material for: A Cross-Sectional Study on the Characteristics of Physical Activity in Pre-Frail Older Adults
Source: Int J Environ Res Public Health. 2021 Nov 24;18(23):12328. doi: 10.3390/ijerph182312328 (PMC8657243; doi:10.3390/ijerph182312328)
Supplement: Supplementary file 1 [file ijerph-18-12328-s001.zip › ijerph-1430120-supplementary.pdf]

Physical Activity Survey (English-translated version)

Q.1. How is your walking condition? Please read each statement and mark the most appropriate response with "○."

1. You can walk normally (you can use a cane)
2. You can walk if you hold on to something
3. You can walk with the support of someone
4. You can't walk

Q.2. How often do you usually sit or lie down in total during the day (do not include sleep time)?

About ______ hours and ______ minutes a day

Q.3. How is your current health? Please read each statement and mark the most appropriate response with "○."

1. Not healthy
2. Not very healthy
3. Fairly healthy
4. Very healthy

Q.4. How often have you had pain somewhere in your body these days? Please mark the most appropriate response with "○."

1. Not at all
2. Slightly
3. Occasionally
4. Mostly
5. Always

Q.5. If you have pain in your body, where do you normally have it? Please mark as many responses as appropriate with "○."

1. Head
2. Neck
3. Shoulder
4. Back
5. Waist
6. Knee
7. Others (   )

Q.6. Have you ever had any of the following illnesses? Please read each statement and mark the most appropriate response with "○."

stroke	No • Yes	1. Under treatment 2. Treated 3. Untreated
hypertension	No • Yes	1. Under treatment 2. Treated 3. Untreated
angina or myocardial infarction	No • Yes	1. Under treatment 2. Treated 3. Untreated
liver disease	No • Yes	1. Under treatment 2. Treated 3. Untreated
diabetes	No • Yes	1. Under treatment 2. Treated 3. Untreated
cancer	No • Yes	1. Under treatment 2. Treated 3. Untreated
osteoporosis	No • Yes	1. Under treatment 2. Treated 3. Untreated
arthritis / arthropathy	No • Yes	1. Under treatment 2. Treated 3. Untreated
mental disorder or mental illness	No • Yes	1. Under treatment 2. Treated 3. Untreated

Q.7. Please circle the answers that apply to you in your recent life.

Do you go out by bus or train by yourself?	YES	NO
Do you go shopping to buy daily necessities by yourself?	YES	NO
Do you manage your own deposits and savings at the bank?	YES	NO
Do you sometimes visit your friends?	YES	NO

Do you turn to your family or friends for advice?	YES	NO
Do you normally climb stairs without using a handrail or the wall for support?	YES	NO
Do you normally stand up from a chair without any aids?	YES	NO
Do you normally walk continuously for around 15 minutes?	YES	NO
Have you experienced a fall in the past year?	YES	NO
Do you have a fear of falling while walking?	YES	NO
Have you lost 2kg or more in the past 6 months?	YES	NO
Do you have any difficulties eating tough foods compared to 6 months ago?	YES	NO
Have you choked on your tea or soup recently?	YES	NO
Do you often experience having a dry mouth?	YES	NO
Do you go out at least once a week?	YES	NO
Do you go out less frequently compared to last year?	YES	NO
Do your family or your friends point out your memory loss?<br>e.g. "You ask the same question over and over again."	YES	NO
Do you make a call by looking up phone numbers?	YES	NO
Do you find yourself not knowing today's date?	YES	NO
In the last 2 weeks have you felt a lack of fulfillment in your daily life?	YES	NO
In the last 2 weeks have you felt a lack of joy when doing the things you used to enjoy?	YES	NO
In the last 2 weeks have you felt difficulty in doing what you could do easily before?	YES	NO
In the last 2 weeks have you felt helpless?	YES	NO
In the last 2 weeks have you felt tired without a reason?	YES	NO

(Working Group on Frailty in JGS)

Q8. Do you currently have a job or any role? Please mark as many responses as appropriate with "○."

1. Working　　2. Doing housework and helping with others
3. Hobbies and volunteers　　4. Farming and gardening
5. Nothing in particular　　6. Others (　　　　　　　　　　　　　　)

Q.9. Is there anything you can enjoy, distract yourself with, or immerse yourself in right now?

1. No　　2. Yes (Specific content:　　　　　　　　　　　　　　)

Q.10. How do you think about people in your area where you live? Please read each statement and mark the most appropriate response with “○”.

	Strongly agree	Somewhat agree	Neither agree nor disagree	Somewhat disagree	Strongly disagree
1. People living in the area help each other.	1	2	3	4	5
2. I can trust people living in my area.	1	2	3	4	5
3. People living in the area greet each other.	1	2	3	4	5
4. If problems occur in the area, people work together to try to resolve the problems.	1	2	3	4	5

Q.11. Are you currently doing the following activities? Please mark as many responses as appropriate with “○.”

1. Local activities (regional groups, neighborhood associations, resident association, women's associations, elderly association associations, youth groups, children's associations, etc.)
2. Sports, hobbies, entertainment activities (various sports, arts and culture activities, lifelong learning, etc.)
3. Volunteer, NPO, Citizen activities (town development, welfare for the elderly, disabled, child-rearing, sports guidance, beautification, crime prevention, disaster prevention, environment, etc.)
4. Other activities (alumni association, social gathering)
5. Not particularly active

Q.12. Do you drink alcohol ? Please mark the most appropriate response with “○.”

1. Almost every day 2. 2-3 times a week 3. About once a week 4. Once a week or less
5. I do not drink

Q.13. Do you smoke cigarettes? Please mark the most appropriate response with “○.”

1. Smoke_________(how many cigarettes do you smoke a day?) 2. I used to smoke but stopped
3. I do not smoke

Q.14-1) How many days per week do you engage in light exercise or calisthenics? Please mark the most appropriate response with “○.”

1. Not at all 2. About one day a week 3. 2-4 days a week 4. Almost every day

Q.14-2) How many days per week do you engage in regular exercise or sport? Please mark the most appropriate response with “○.”

1. Not at all 2. About one day a week 3. 2-4 days a week 4. Almost every day

Q.15-1) Please tell us your name and sex.

name		sex	Male • Female

Q.15-2) Please tell us your date of birth and your age as of September 1, 2017.

date of birth		age	

Q.15-3) Which of the following is your final academic background? Please mark the most appropriate response with "○."

1. Until junior high school 2. High school 3. Junior college / vocational school
4. University / graduate school

Q.16. Please tell us your current address and phone number.

address	
phone number	

Q.17-1）Which of the following is your current residence? Please mark the most appropriate response with "○."

1. Owned house 2. Rented house or apartment 3. Temporary housing
4. Housing constructed after a disaster 5. Home of a friend/relative 6. Other ( )

Q.17-2）How many times have you moved since the Great East Japan Earthquake? Please mark the most appropriate response with "○."

1. Not at all 2. 1 time 3. 2 times 4. 3 times or more

Q.18. Who do you currently live with? As an answer to the next question, please mark the most appropriate response with "○."

1. Single household (by myself) 2. Married couple only household
3. Married couple and unmarried children household
4. Third generation family 5. Myself and unmarried children 6. Other

Q.19. Are you currently certified for long-term care insurance? Please mark the most appropriate response with "○."

1. Not certified
2. Certified (Requiring help: , Long-term care level:)
3. Currently applying or planning to apply

That's the end of our question. Thank you for your cooperation in the questionnaire.

（This questionnaire was originally written in Japanese.）
